# Biotherapeutics: Challenges and Opportunities for Predictive Toxicology of Monoclonal Antibodies

**DOI:** 10.3390/ijms19113685

**Published:** 2018-11-21

**Authors:** Dale E. Johnson

**Affiliations:** Morgan Hall, University of California, Berkeley, Berkeley, CA 94720, USA; daleejohnson@berkeley.edu; Tel.: +510-316-1197; Fax: +866-803-8811

**Keywords:** monoclonal antibodies (mAbs), immunogenicity, anti-drug antibody (ADA), cytokine release, acute phase reactions, immune complex assays, biomarkers

## Abstract

Biotherapeutics are a rapidly growing portion of the total pharmaceutical market accounting for almost one-half of recent new drug approvals. A major portion of these approvals each year are monoclonal antibodies (mAbs). During development, non-clinical pharmacology and toxicology testing of mAbs differs from that done with chemical entities since these biotherapeutics are derived from a biological source and therefore the animal models must share the same epitopes (targets) as humans to elicit a pharmacological response. Mechanisms of toxicity of mAbs are both pharmacological and non-pharmacological in nature; however, standard in silico predictive toxicological methods used in research and development of chemical entities currently do not apply to these biotherapeutics. Challenges and potential opportunities exist for new methodologies to provide a more predictive program to assess and monitor potential adverse drug reactions of mAbs for specific patients before and during clinical trials and after market approval.

## 1. Introduction

Biotherapeutics or biologicals are drug therapy products where the active substance is extracted or produced from a biological source [1,2]. These products include recombinant proteins and hormones, monoclonal antibodies (mAbs), cytokines, growth factors, gene therapy products, vaccines, cell-based products, gene-silencing/editing therapies, tissue-engineered products, and stem cell therapies [1,2,3,4,5,6]. Many of the biotherapeutic molecules in development or recently approved are mAbs and these are considered the most rapidly growing drug class in oncology, anti-immunity, and chronic inflammatory diseases. Monoclonal antibodies act therapeutically through multiple mechanisms including apoptosis in cells that express the target (antigen), by blocking targeted molecular functions, and/or by modulating signaling pathways [2]. Functionally, when the Fab (fragment antigen binding) part of the mAb binds to its antigen target, it blocks the antigen interaction with a ligand. The mAb can also elicit actions through the Fc (fragment constant) region, which includes antibody-dependent cell-mediated cytotoxicity (ADCC) and antibody-dependent cellular phagocytosis [2]. The subclasses of IgG antibodies include IgG1 and IgG3 which are the most active; they fix complement, bind to Fc receptors on phagocytes, and elicit ADCC. However, IgG3 is very seldom used for therapeutic mAbs as the hinge region is prone to proteolysis which results in a decreased half-life [2]. IgG2 mAbs fix complement moderately and have low affinity to bind to Fc receptors. IgG4 mAbs bind to Fc receptors, however they do not fix complement and there is no ADCC. The hinge region is also susceptible to the in vivo formation of bispecific antibodies, and must be mutated to avoid this. Therefore, IgG1 mAbs are the most common subclass used for oncology [1]. Several structural modifications have been made to increase therapeutic efficacy and potentially reduce side effects. These include: targeting immunomodulatory molecules (cytokines) via bispecific antibody fragments and/or scFv (single chain variable fragments or Ab-ligand fusion proteins) to tumor cells to induce apoptosis; IL-2 fusion proteins; antibody drug conjugates (ADCs); and antibody-directed enzyme prodrug therapy (ADEPT) by directly targeting enzymes to the tumor cell and delivering a prodrug that is converted to a chemotherapeutic by the enzyme targeted [2,3].

## 2. New mAb Approvals

**Figure 1:**
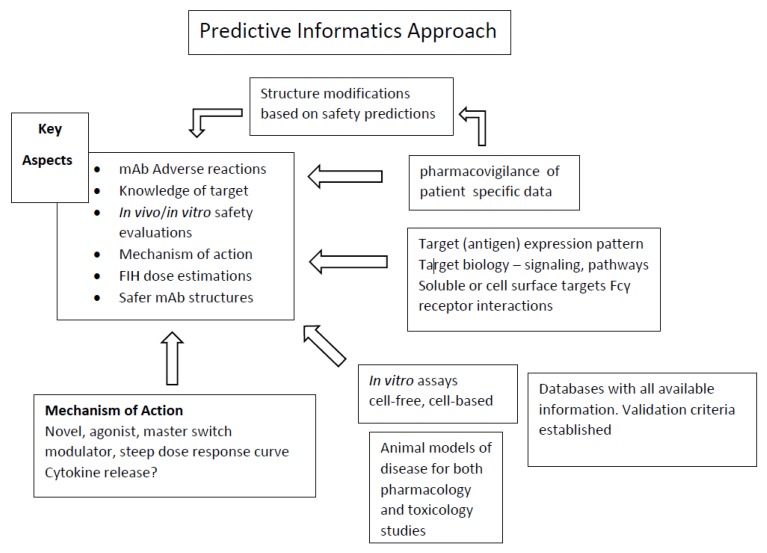
Informatics Approach for Predictive Toxicology of Monoclonal Antibodies. Informatics processing of several data sources to create a predictive tool for identifying Key Aspects during mAb research and development.

As an example of the new approvals, 46 total drugs were approved by United States Food and Drug Administration (USFDA) in 2017; 24 were chemical entities and 22 were biotherapeutics. Of the 22 biotherapeutics, 10 were mAbs. These included brodalumab, dupilumab, sarilumab, guselkumab, bentalizumab, ocrelizumab, inotuzumab, avelumab, duvalumab, and emicizumab [7]. Table 1 lists these with names, target, mechanism of action, and adverse reactions reported in clinical trials. At the time of this review, through 14 September 2018, eight additional mAbs were approved by USFDA. These included fremanezumab–vfrm, moxetumab pasudotox–tdfk, lanadellumab, mogalmulizumab kpkc, erenumab–aooe, burosumab–twza, tildrakizumab, and ibalizumab–uiyk [8]. These are listed in Table 2. As can be seen, with the latest approved mAbs as compared to previously approved mAbs [2,9], the adverse effects continue to be similar year to year, and in several cases are severe in nature with a high dependence on the patient population under consideration. Sim and colleagues [10], reporting on a multi-year pharmacovigilance study of patients receiving mAbs in Korea, found that severe adverse reactions developed more frequently in children (<12 years) and in the elderly (≥65 years), and anaphylaxis was not rare in these age groups. As is highlighted in this review, there are definite concerns whether a “real” predictive toxicology program can be instituted as with chemical entities; however, based on new methodologies and bioinformatics, a scheme can be envisioned, which is highlighted in Figure 1. The key aspects include: understanding adverse effects of mAbs; nonclinical safety evaluation both in vivo and in vitro; first-in-human (FIH) dose estimations; and defining “safer” mAb structures for design and replacement efforts.

## 3. Adverse Effects of mAbs

Targeted mAbs for cancer therapy are typically IgG1 Fc design with immunomodulatory function [1,2]. Many of the immunomodulatory effects of mAbs are desirable, classified as intended immune pharmacology, which is required for clinical efficacy. However, activation or suppression/depletion of non-target immune cells and mediators, permanent non-reversible changes to immune target cells/pathways, or any unintended effects associated with the intended pharmacology such as severe infusion reactions, cytokine release syndrome (CRS), cell and tissue injury, inflammation, infection and cancer are considered immune toxicity [11]. Adverse effects of mAbs are common in patients and these are either mediated through the intended pharmacological action or are nonpharmacological. Pharmacological action is defined as the interaction of the biotherapeutic with its intended target, such as the binding of anti-vascular endothelial growth factor (anti-VEGF) antibody to VEGF, hypoglycemia from insulin, and infections related to the use immunomodulators. Nonpharmacological adverse effects are those unrelated to the interaction with the intended target which can include hypersensitivity reactions secondary to an immune response or acute phase reactions due to the Fc region of a mAb [12]. Demlova and colleagues [13] classified these adverse effects as: (A) high cytokine and cytokine release syndrome as with anti-CD3; (B) hypersensitivity, both immediate as with IgE-mediated, and delayed as with IgG- and T cell-mediated; (C) immune or cytokine imbalance syndromes, not explained by high cytokine levels or typical hypersensitivity reactions; (D) cross-reactivity, such as reaction with normal cells; and (E) non-immunological side effects. The more recent development of immune checkpoint inhibitors, such as those targeting cytotoxic T-lymphocyte antigen 4 (CTLA-4) and programmed cell death-1 (PD-1), are associated with immune-related adverse events leading to T-cell inflammatory infiltration of solid organs, and increased serum inflammatory cytokines. Frequent side effects include dermatologic, gastrointestinal, hepatic, endocrine, and other inflammatory events [11]. Kizhedath and colleagues [9] highlighted the side effects of approved mAbs with an extensive list including mAb names, type of Ab or derivative, target antigen, indication, and reported adverse effects. As an example, mAb-derived therapeutics where infections have been a side effect include the following types: fusion proteins, IgG1κ, IgG1λ, Fab’-GIκ, IgG2, IgG2/G4κ, IgG4, and CP (composite protein). The antigens targeted by mAb therapeutics where infections have been side effects include: TNF, TNFSF13B, VEGFA, TNFRSF8, ILIB, CD80-CD86, TNSF11, C5, PCSK9, Dabigatran etexilate mesylate, IL5, ITGA4, MS4A1, IGHE, anthrax protective antigen, MPL, IL6, IL6R, EI, and ITGB7. Importantly, the antigens targeted by therapeutic mAbs are not always expressed exclusively at the disease sites [11] and systemic injection of therapeutic mAbs may cause considerable adverse effects which can decrease treatment efficacies. Chen and colleagues [14] highlighted this with examples such as epidermal growth factor receptor (EGFR) which is over-expressed in some tumors and has an important role in tumor progression. Importantly, EGFR is also expressed in some epithelial cells and systemic administration of anti-EGFR mAbs will induce adverse effects such as skin rash in a majority of patients with metastatic colorectal cancer. In another example, tumor necrosis factor-α (TNF-α) is a pro-inflammatory cytokine over-expressed in the joints of rheumatoid arthritis (RA) patients. Importantly, TNF-α is also an important cytokine in the defense against microbial infections. Therefore, as Chen and colleagues [14] pointed out, systemic targeting of TNF-α with anti-TNF-α mAbs such as Remicade and Humira is known to create a higher risk of serious infections, and long-term treatment with Remicade may increase lymphoma incidence [14]. Several of the immune-mediated adverse effects, which include hypersensitivity reactions, abrogation of the intended pharmacologic effect, and altered pharmacokinetic profiles, which impact therapeutic exposure of the mAb, are due to anti-drug antibodies (ADAs) that generally result in the formation of the drug/ADA immune complex (IC). The enhanced clearance results from recognition of ICs by Fc receptors (FcR). ADAs can also lead to cross-reactivity to endogenous versions of the protein and significant adverse effects, ranging from infusion reactions to anaphylactic reactions [15]. Some of these adverse effects are patient specific due to the disease being treated, co-morbidities, and concurrent therapies. This is an example of why adverse effects from mAbs must also include patient specific information, as many mAb treatments are part of a personalized medicine approach, which would require stratification of patients with regard to how they respond or do not respond, including in each case whether adverse reactions did occur and to what extent [16].

## 4. Nonclinical Safety Evaluations for mAbs

The main objectives of the nonclinical evaluation of biotherapeutics are: (1) identification of target organs for toxicity and to determine whether the toxicity is reversible after the treatment has stopped; (2) identification of a safe starting dose for human Phase I clinical trials and subsequent dose escalation schemes, which is highly dependent on the patient population for the Phase I trial(s); and (3) provide information to monitor safety parameters in clinical trials [5,17,18,19,20]. Key factors that must be considered for mAb development are: knowledge of the antigen target biology and location of the target, both desired and undesired. Additional factors include pharmacological properties and mechanism of action of the therapeutic mAb; exposure–response relationships; estimates of pharmacokinetic parameters and how these may relate to the determination of a recovery period; and clearly defined clinical trial design and potential characteristics and co-morbidities of patients [5]. In the development process, non-clinical studies rarely identify toxicities that are dose limiting and the selection of a pharmacologically relevant species is of paramount importance [4,5,6,17,18,19,20]. Accordingly, the lack of toxicity related to significant decreased or a lack of pharmacological action in the model negates the use of lack of toxicity to be used as a measure of safety. For mAbs, the most relevant toxicology species is the cynomolgus monkey. Iwasaki and colleagues [21] recently reviewed animal species that were used for non-clinical assessments and that cross-reacted to 39 approved mAbs drugs. Their analysis showed that cynomolgus monkeys were the most frequently used species in non-clinical studies of marketed mAbs and this species cross-reacted with 16 out of 18 anticancer mAbs surveyed and 16 out of 21 non-cancer mAbs. Cancer related cross-reacting targets included: CCR4, CD20, CD30, CD52, CTLA-4, EGFR, HER2, PD-1, VEGF and VEGFR-2. Non-cancer related cross-reacting targets included: CD25, IgF, IL-5, IL-12/23p40, IL-17A, IL-17RA, IL-6R, α4-integrin, PCSK9, RANKL, TNFα, VEGF-A, and CD. Of interest from a safety standpoint is the history of mAbs targeting T cells or engaging T-cell receptors on T lymphocytes which stimulated activation, proliferation and the release of cytokines and chemokines. This has stimulated the still on-going investigation into why certain patient populations are more susceptible [5,6,10]. Examples include the TGN1412 incident [22] with direct stimulation of T cells and a resulting cytokine storm; and muromomab and OKT3, resulting in significantly increased TNF-α serum levels [6]. Other examples include Campath H-1 targeting CD52 on lymphocytes and monocytes with resulting elevated levels of TNF-α, IFN- ү, and IL-6; rituximab (anti-CD20) resulting in rapid increases in TNF-α and IL-6; and visilizumab (anti-CD3) induced cytokine release syndrome resulting in liver injury [6]. The TGN1412 cytokine storm in the Phase I trial in normal volunteers was highly publicized and eventually led to a change in the dose calculations for First-in-Human trials, which is discussed below in this review. Agonist molecules, such as TGN1412 as opposed to neutralizing or function-blocking mAbs, only need to occupy 10–20% of available receptors to elicit a maximum pharmacological effect, whereas, with neutralizing or function-blocking mAbs, approximately 80–100% receptor occupancy (RO) is required to elicit a maximum effect [6]. This can represent a significant case with immunomodulatory and agonist molecules and require different approaches when extrapolating pharmacokinetic/pharmacodynamics (PK/PD) data from the standard non-clinical species. The complication is that certain lymphocyte subsets are different in cynomolgus monkeys compared with those found in humans. A striking difference in peripheral T cells between humans and cynomolgus monkeys is the substantial presence of CD4+/CD8+ (double-positive) T-cell phenotype in cynomolgus monkeys [23]. Double- positive T cells exhibit a resting memory phenotype that increases proportionally with age. CD28 and CD29 surface antigens also change in relation to age. Teroa found a difference in function of CD28 in relation to T-cell activation and cytokine release between young and adult cynomolgus monkeys [24]. Since young monkeys (<3 years of age) are typically used in toxicology studies, the actual T-cell phenotype becomes an important issue. It is probable that the reactivity or sensitivity to an agonist antibody might be lower in young monkeys compared with adult monkeys [6,23]. This is a relevant question as it relates to predictive toxicology in the non-clinical animal species.

Brennan and Kiessling [11] provided a detailed review of adverse reactions to mAbs and nonclinical strategies including in vitro studies used to both predict and confirm the reactions in the nonclinical animal models as well as in patients. The overall strategy includes: 

(A) In silico review of the target biology and consequence of modulation from studies from knockout (KO) mice and humans or any available data with mAbs or other drugs against the same target or pathway. This would include pharmacovigilance studies where details are included in accessible databases [10,25,26,27,28,29,30,31]. 

(B) Rational design of mAb candidates to remove any unwanted pharmacological effects such as structure of mAbs intended to suppress the immune system and/or selection of mAbs with low/no Fc effector function to avoid interaction with FcγRs on immune cells. For example, IgG4 with naturally low effector function or Fc-engineered IgG1 with low/no Fc effector function and/or use of Fab fragments would avoid receptor cross-linking [27,32,33].

(C) In vitro and ex vivo studies with human/animal cells and tissues to assess both on-target and off-target binding, pharmacological activity and immunogenicity potential. Tissue cross-reactivity (TCR) studies use in vitro Immunohistochemistry (IHC) techniques to characterize mAb binding to antigenic determinants in frozen human tissues and animal tissues from pharmacologically-relevant species. These techniques can be used to assist in the interpretation of any pathology-related findings toxicology animal toxicology studies as compared to the relevance of any observed binding in the human tissue panel [34,35,36,37,38,39].

(D) In vivo studies in pharmacologically-relevant species(s). Data from studies in Points (C) and (D) would theoretically provide an understanding of expected human immune related pharmacological and potential toxicological effects of a mAb and whether this is likely to be predicted by toxicology species. Several other assays have been developed [40,41,42,43]; however, as described in several reviews, many of the more serious adverse events observed in humans when administered mAbs are classified as rare events such as cancer, serious infections, and progressive multifocal leukoencephalopathy. Therefore, these serious events are unlikely to be derisked from a mAb by nonclinical in vitro and in vivo studies [11].

## 5. First-in-Human (FIH) Dose Calculations

The science and regulatory aspects of FIH dose selection for mAbs have evolved into a target mechanism-based model which utilizes exposure–response relationships from both in vitro and in vivo studies [6]. Important aspects include the minimally effective exposure concentrations for: biological activity levels (e.g., ED_10_; pharmacologically active levels (e.g., ED_50_); the highest safe dose (NOAEL) established in toxicology studies and/or toxicodynamic end point estimates from toxicology studies; and exposure- or concentration-related biomarker changes both in vitro and in vivo. The ED_50_ calculations are particularly important for FIH cancer trials [6] as a pharmacologically active dose must be used in these patients. Another important concept is the minimal anticipated biological effect level (MABEL) and its consideration in the selection of a safe maximum recommended starting dose for (MRSD) in humans. This is particularly important for mAbs considered to be high risk molecules such as agonists, as discussed in detail by Brennan and Kiessling [11]. The MABEL represents the lowest dose or concentration required to produce pharmacological activity in vivo and/or in vitro in animal/human systems. The MRSD is selected based on demonstration of an adequate safety margin compared with doses which cause toxicity in the nonclinical animal studies, or the NOAEL established in toxicology studies. The NOAEL is used in the case of mAbs with anticipated or low toxicity, as well as consideration of the MABEL. The use and calculation of the MABEL for mAbs and should utilize all relevant biological and pharmacological information. Key considerations defined by Brennan and Kiessling [11] for estimating the most relevant FIH dose include:

(A) The nature and duration of the pharmacological effect(s) or mode of action (MOA). Is the mAb an agonist with strong immune cell activation and cytokine release potential or is it an antagonist? 

(B) The novelty of the molecule. Is it a standard IgG or mutivalent and/or highly engineered construct?

(C) How relevant or sensitive are the nonclinical models to assess and/or predict safety of the pharmacological effect of the mAb (dependent on relative target binding and in some cases FcγR binding, target distribution and expression level and pharmacological activity between animals and humans)?

Ideally, these parameters are measured in the in vivo and/or in vitro studies, but frequently they are predicted via modeling or allometric extrapolation. This is the standard case for estimated human doses and/or exposures [6]. The in vivo elimination of mAbs occurs through antigen-mediated clearance (CL) or binding to the intended target, also referred to as the “ antigen sink”. Additionally, clearance occurs through binding interactions with nonspecific Fc receptors in the reticuloendothelial system and FcRn (neonatal or salvage receptors), also referred to as “catabolism” mechanisms. When antigen-mediated elimination is not saturated, nonlinear, concentration-dependent CL is expected. At low doses, CL will be faster and the half-life will be lower than at higher saturating doses. Linear elimination (assuming nonspecific CL approximates endogenous IgG) occurs at higher doses with antigen saturation, with slower CL and longer half-life [44,45,46]. Ling and colleagues conducted a study of interspecies scaling with data from 14 mAbs, suggesting a rational approach to use a single species for allometric scaling to human CL [46]. The authors concluded that simplified allometric scaling using a fixed exponent will provide accurate predictions using the following equation: CL_human_ = CL_animal_ × (BW_human_/BW_animal_)^b^, where b is 0.85 or 0.9. This represents a single-species allometric scaling with mAbs for FIH dose estimation. Based on the target patient population, safety factors such as a 10-fold reduction in the FIH dose calculation, may be used to start a dose escalation scheme.

## 6. mAb Structure and Predictive Safety/Toxicity

Antibody therapies with high functional efficiency and potential low toxicity are becoming one of the major approaches in mAb therapeutics [47]. Based on high-throughput sequencing and increased availability of experimental structures of antibodies/antibody-antigen complexes, computational approaches are now being used to predict antibody/antigen structures, engineer the function of antibodies, and design antibody–antigen complexes with improved properties [47]. However, the field of in silico prediction of adverse effects in humans from mAb structures is still in early stages. The standard methodology used for in silico predictive toxicology in drug development, such as quantitative structure–activity relationships (QSAR), is based on connecting an endpoint (toxic activity) to descriptors of chemical structures from compounds that either induce the toxic activity or do not. Chemical descriptors can be derived from physiochemical, structural, electronic, or steric parameters. Large datasets are used to derive and form the basis of accuracy for the predictions, which could include toxicities ranging from hepatotoxicity to genotoxicity and potential carcinogenicity. Zhao and colleagues [47] summarized recent progress in the field of in silico design of antibodies, including antibody structure modeling, antibody–antigen complex prediction, and antibody stability evaluation. Allosteric effects in antibodies and functions force fields are becoming more accurate to model molecular behaviors, especially local rearrangement. Thus, Zhao and colleagues [47] reported that in silico molecular modeling techniques are becoming more popular to engineer antibodies such as Fc-based antibody domains and fragments [48], disulfide bonds [49], and T-cell receptor (TCR) mimic antibodies [50] with desired properties. These properties include viscosity and phase separation [51]. They also suggested that an alternative computational approach to physical modeling is the knowledge-based residue pair preference on epitope–paratope interfaces [47]. With the increasing availability of crystal structures of antibody–antigen complexes in the Protein Data Bank (PDB), a statistical amino acid interaction preference matrix can be used to predict the antibody–antigen recognition [47]. As discussed by Kizhedath and colleagues [9], the IMGT mAb database [52] provides comprehensive information on structure, primary sequences, developmental status, targets and documents relating to mAb approvals. Descriptors for proteins molecules can be generated by different software such as PseAAC, Protein Recon, PROFEAT and ProtDCal. ProtDCal has the capacity to generate a higher number of non-redundant of molecular descriptors for proteins from FASTA or PDB files [53]. There are different modeling platforms for predicting antibody structures from primary sequences such as PIGS (Prediction of Immunoglobulin Structures), Rosetta antibody, Web Antibody Modelling (WAM) and Abysis databases [54]. RCSB integrates different bioinformatics and structural tools for comparison of primary and secondary structures. Advances made in Proteochemometric (PCM) modeling techniques include a new descriptor for antigen–antibody interaction called epitope–paratope interaction fingerprint (EPIF) which allows for the simplification the antigen–antibody interaction. Different from QSAR modeling, PCM contains both ligand and target descriptors to correlate with activity data [55]. Olimpieri and colleagues [56] developed prediction of Antibody Contacts (proABC), a web server for predicting which residues of an antibody are involved in recognizing its targeted cognate antigen. The technology is based on a machine-learning method trained on sequence and sequence-derived features. Using the antibody sequence, proABC estimates the probability that each residue in its sequence interacts with the cognate antigen. Three different types of interaction are predicted separately (hydrogen bond, hydrophobic and other non-bonded interactions). The results allow a comprehensive examination of the residues that could directly interact with the antigen. proABC also builds a 3D model of the antibody, in which residues are coded according to their contact probability [56]. Di Rienzo and colleagues [57] described a superposition free method for comparing the surfaces of antibody binding sites which can be used to both compare and cluster sets of antibodies. These antibody clusters provide information about the nature of the bound antigen and when combined with the prediction of the number of direct antibody antigen contacts, allows for the discrimination between protein and non-protein binding antibodies. This technology would be relevant in several aspects of antibody science, such as to select the framework to be used for a combinatorial antibody library. The concept of a combinatorial library for antibodies takes on added significance based on the advances made in phage display production of mAbs. As described by Clementi et al. [58] and Frenzel et al. [59], the phage display method is based on a physical link between function (antigen binding) and information (antibody gene) in a nanoparticle (the phage virus particle). The antigen binding parts of the antibodies, either as Fab (fragment antigen binding) [60,61] or scFv (single chain fragment variable) [62], are genetically linked to the surface protein III (pIII) of the M13 phage and thus expressed on the surface of the virus particle. Mixtures of such phage particles, each encoding and presenting a different antibody can be produced and these mixtures contain billions of different individual clones, allowing to mimic the entire naive antibody repertoire. With these antibody libraries, the genes encoding specific antibodies which can bind to the antigen of interest can be selected by affinity enrichment on the antigen in vitro (“panning”). These advancements have further enhanced the ability to predefine properties of antibodies prior to the production phase. In another aspect of predefining potential toxicity from mAbs, Mukherjee and colleagues [63] used molecular protein docking in analyzing potential reasons certain types of adverse reactions are more prevalent with one mAb than with another similar mAb. The authors used the Hex open source software with two mAbs, bevacizumab and tratuzumab, into the VEGF receptor, the Her-2/Neu receptor, and the dopamine-2 receptor with an analysis of binding affinity to suggest why the symptom of nausea is more pronounced with bevacizumab [63]. 

As another example of reducing potential on-target toxicity from mAbs, Chen and colleagues [14] engineered a protease-activated pro-antibody that can be selectively activated in the region of disease sites to provide specific localization of the therapeutic antibody. The pro-antibody strategy involves masking the antibody binding sites by inhibitory domains derived from latency-associated peptide (LAP) of transforming growth factor-β (TGF-β) and C2b of complement factor 2 and CBa of complement factor B, through a substrate peptide (GPLGVR) for matrix metalloproteinase-2 (MMP-2), to the heavy chain of the antibody [64]. Through this approach, the inhibitory domains were expected to block the binding activity of the pro-antibody until MMP-2 activation. Inhibitory domains are based on two principles: first, the sequences must come from endogenous proteins such that the probability of provoking anti-inhibitory domain immune responses is minimized; and, second, the inhibitory domains must not display apparent or known biological function other than blocking the activity of the original proteins. Chen and colleagues [14] indicated that masking antibody binding sites may be an effective way to prevent or reduce adverse effects during monoclonal antibody therapy by allowing antibody binding to antigens at disease sites (protease positive) but not in normal tissues (protease negative). 

## 7. Conclusions

An overall predictive toxicology program for mAbs in research and development to lessen or mitigate serious safety concerns is still considered to be in an early stage [65], although key components (see Figure 1) are in mid- to late-stage development. This includes advances in: in silico predictions [25,26,27,28,29,30,31,66] and different mAb structure and/or functional designs [27,32,33,67]. In vitro assays are fully functional [34,35,36,37,38,39,40,41,42,43]; however, the obvious variability that occurs (tissue-to-tissue, assay formats, and accuracy standards) has to be considered, particularly when using an in vitro endpoint in an in vitro/in vivo extrapolation (IVIV) such as in a pharmacokinetic/pharmacodynamic (PK/PD) modeling and analysis. The key components moving forward are the variability in outcome based on patient characteristics, disease status, and concomitant drug therapy. A significant area for more innovation is in the field of immunotherapy, where severe infections [68] and alterations in an individual’s PK [69,70] can occur and significantly affect the outcome of the intended therapy. The potential for identifying the location(s) of patient specific targeted epitopes [71] in standard laboratory assays such as in a blood draw would be an ideal pursuit for diagnostic entities. The concept of FIH dose calculations [72] should be expanded to include biomarkers that would identify patient specific outcomes as they relate to both the intended mechanism of action and possible or probable safety issues. On the non-clinical side, this would probably be derived from a disease model in a humanized animal model. The dosing scheme would include a complete understanding of receptor occupancy for each individual while undergoing continued therapy, and how this may change the dosing scheme. As mentioned, these components are all in various stages of development and an informatics solution as outlined in Figure 1 for tying everything together is the obvious next step. The key aspects of a predictive toxicology informatics approach for mAbs are a complete knowledge of the target and on- and off-target interactions leading to potential toxicity; understanding and documenting adverse reactions based on patient specific criteria; understanding mAb structural features and how to modify structures to maintain therapeutic efficacy and mediate safety concerns; selection of the most relevant in vitro and in vivo models for safety and efficacy; and creation and maintenance of high content databases geared toward all aspects of mAb research and development.

## Figures and Tables

**Table 1:** USFDA mAb Approvals in 2017.

mAb	Target(s) Mode of Action	Indication(s)	Adverse Effects
Brodalumab (Siliq) Valeant Pharmaceuticals	IL-17RA antagonist	Moderate to severe plaque psoriasis	Severe infections, suicidal and behavior ideation
Dupilumab (Dupixent) Regeneron Pharmaceuticals	IL-4 Rα antagonist Inhibits IL-4 and IL-13 signaling	Moderate to severe atopic dermatitis	Injection site reactions, infections, conjunctivitis, blepharitis, keratitis, eye pruritus, dry eye
Serilumab (Kevzara) Sanofi	IL-6 R antagonist	Moderately to severe active rheumatoid arthritis	Hearing deficiency, potential for severe opportunistic infections, neutropenia, increased ALT, injection site erythema,
Guselkumab (Tremfya) Janssen Biotech	IL-23 inhibitor Inhibits the release of pro-inflammatory chemokines	Moderate to severe plaque psoriasis	May increase risk of infections, headache, injection site reactions, arthralgia, diarrhea, gastroenteritis, tinea infections, herpes simplex infections
Benralizumab (Fasenra) Astra Zeneca	IL-5 R binding on eosinophils; attracts Natural Killer Cells to induce apoptosis	Severe asthma with an eosinophilic phenotype	Headache, pharyngitis, pyrexia, hypersensitivity reactions
Ocrelizumab (Ocrevus) Genentech	CD20 antigen on B lymphocytes ADCC and complement lysis	Multiple sclerosis	Upper respiratory infections, infusion reaction
Inotuzumab ozogamicin (Besponsa) Pfizer	CD-22 directed ADC with N-acetyl-gamma-calicheomicin	Relapsed or refractory B-cell precursor acute lymphoblastic leukemia	Hepatotoxicity, veno-occlusive disease or sinusoidal obstruction syndrome; thrombocytopenia; neutropenia; infection; anemia; leukopenia; fatigue; hemorrhage
Avelumab (Bavencio) EMD Serono /Pfizer	PD-L1–blocks interaction between PD-L1 and PD-1 and B7-1	Metastatic Merkel cell carcinoma	Fatigue, musculoskeletal pain, diarrhea, nausea, infusion-related reaction, rash, decreased appetite, peripheral edema
Durvalumab (Imfinzi) Astra Zeneca	PD-L1–blocks T-cell function and activation via PD-1 and CD80	Advanced or metastatic urothelial carcinoma and Stage III non- small cell lung cancer	Fatigue, musculoskeletal pain, constipation, infections, peripheral edema, decreased appetite
Emicizumab–kxyh (Hemlibra) Genentech	Bispecific factor IXa and factor X directed–to restore missing factor VIII	Prevention or reduction of bleeding episodes in patients with Hemophilia A	Thrombotic microangiopathic and thrombic effects, injection site reactions, headaches

**Table 2:** USFDA mAb Approvals through 3Q 2018.

mAb	Target(s) Mode of Action	Indication(s)	Adverse Effects
Fremanezumab–vfrm (AJOVY) Teva Pharmaceuticals	Anti-CGRP	Migrane	Injection site reactions, allergic reactions
Tildrakizumab (Ilumya) Sun Pharmaceuticals	Binds to IL-23 and inhibits interaction with IL-23 R; inhibits release of proinflammatory cytokines and chemokines	Moderate to severe plaque psoriasis	Upper respiratory infections, injection site reactions, diarrhea
Ibalizumab–uiyk (Trogarozo) Tai Med Biologics	CD4 directed HIV-1 inhibitor; blocks HIV-1 from infecting CD4 + T cells	HIV-1 infection	Diarrhea, dizziness, nausea, rash, immune reconstitution syndrome
Burosumab–twza (Crysvita) Ultragenyx	FGF 23 blocking mAb	x-linked hypophosphatemia	Headache, injection site reaction, vomiting, pyrexia, pain in extremity, vitamin D decreased, blood phosphorus increased
Erenumab–aooe (Aimovig) Amgen	CGRP receptor antagonist	Preventative treatment of migraine	Injection site reactions, constipation
Mogamulizumab kpkc (Poteligeo) Kyowa Kirin Incc	Binds to CCR4	Relapsed or refractory mycosis fungoides or Sẽzary syndrome	Rash
Lanadelumab (Takhzyro) Shire	Plasma kallikrein inhibitor	Prevention of Type I and II hereditary angioedema (HAE)	Injection site reactions, upper respiratory infections, headache, rash, muscle pain, dizziness, diarrhea
Moxeturmomab psuedotox–tdfx (lumoxiti) Astra Zeneca	CD-22 cytotoxin	Refractory hairy cell leukemia	Hypertension, febrile, neutropenia, hemolytic uremic syndrome, capillary leak syndrome

## Abbreviations

ADA	anti-drug antibodies
ADC	antibody drug conjugate
ADCC	antibody-dependent cell-mediated
ADEPT	antibody-directed enzyme prodrug therapy
CCR	complex chromosomal rearrangement
CD	cluster of differentiation, which identifies cell surface molecules
CRS	cytokine release syndrome
CTLA-4	cytotoxic T-lymphocyte antigen 4
EGFR	epidermal growth factor receptor
Fab	fragment antigen binding
Fc	fragment constant
FIH	First-in-human
IC	immune complex
IgG	immunoglobulin G
IHC	Immunohistochemistry
mAb	monoclonal antibody
MABEL	minimal anticipated biological effect level
MRSD	maximum recommended starting dose
NOAEL	no observable adverse effect level
PD-1	programmed cell death-1
PK/PD	pharmacokinetic/pharmacodynamic
scFv	single chain variable fragments
TCR	tissue cross-reactivity
TNF	tumor necrosis factor
USFDA	United States Food and Drug Administration
VEGF	vascular endothelial growth factor
